# Distinguishing mechanisms of adverse drug reactions from mechanisms of actions of drugs

**DOI:** 10.18632/oncotarget.25968

**Published:** 2018-08-21

**Authors:** Ting-Hui Wu, Nagla Karim, Jeffrey A. Moscow

**Affiliations:** Jeffrey A. Moscow: Department of Investigational Drug Branch, National Cancer Institute, Bethesda, MD, USA

**Keywords:** chemobrain, doxorubicin

Chemotherapy-induced cognitive impairment (CICI) is a prevalent complication of anti-cancer therapy among cancer survivors. In the past decade, longitudinal studies assessing the pre- and post-chemotherapy cognitive functions of cancer patients have demonstrated the impact of CICI on physical health and quality of life [1]. Given the importance of the issue, there has been a growing interest in studying the underlying mechanisms and corresponding treatments of CICI. One of the dilemmas of CICI is that agents known to be associated with it, including doxorubicin, do not cross the blood-brain barrier. How then can these agents have a deleterious effect on the central nervous system?

In this issue of Oncotarget, Keeney et al. describe an intriguing animal model of doxorubicin-induced CICI, one both addresses the CNS penetration dilemma and one that also proposes a potentially treatable mechanism of CICI [2]. In this model, doxorubicin-induced plasma protein oxidation affects cognitive function by stimulating TNF-α, which is the intermediary that crosses into the CNS and subsequently affects phosphatidylcholine levels by inhibiting phosphatidylcholine-specific phospholipase C. The authors also demonstrate that the antioxidant MESNA can alleviate these adverse biological effects caused by doxorubicin; this finding further supports the concept that the initial CICI chain-of-events is an extracellular oxidation reaction, unrelated to the anticancer mechanism of action of doxorubicin, so one could envision a mechanism-based therapy to prevent CICI without diminishing the effectiveness of doxorubicin chemotherapy.

There are several other proposed mechanisms of CICI, involving altered cytokine regulation, damage of DNA, and attenuation of neuronal repair directly or indirectly-related to chemotherapy [3]. Dysregulation of the immune function has been postulated to play a key role in the predisposition or perpetuation of CICI. The cytokines IL-1, IL-6, IL-8, and MCP-1, in addition to TNF-α, have been reported to be associated with chemotherapy-induced deficits in cognitive performance [4, 5]. Certain genetic polymorphisms or mutations associated with neuronal repair (*APOE* E4 allele, *BDNF* Val66Met), DNA repair (*OGG1, APEX1, XRCC1*), telomere length (*DDX11*) and neurotransmitter activity (*COMT* Val158Met) could also lead to higher susceptibility to CICI [6-9].

Despite the advances in CICI research, there are many gaps between laboratory, epidemiological studies and clinical practice to be filled. The roles of cancer itself, chemotherapy and the interaction between the two in CICI are not well understood. Some cancer patients who never receive chemotherapy experienced similar cognition impairment to those with CICI [10, 11], and many studies on CICI lack a non-chemotherapy control group [12]. A proportion of CICI cases had persistent or delayed onset of cognitive deficit. The mechanism of CICI in these patients is obscure since most animal models focused on the short-term effect of chemotherapy [3]. Furthermore, the majority of CICI researches focused on breast cancer and the chemotherapy regimens used in the disease. Understanding CICI beyond breast cancer will help us determine whether the mechanism and risk of CICI differ among populations or treatment received [12].

The National Cancer Institute (NCI) recognizes that further understanding of the mechanisms of all treatment-related adverse events, including CICI, has the potential for improving the quality of life for an ever-increasing number of cancer survivors. Most significantly, NCI has challenged the research community to address important issues in cancer research through its provocative questions initiative; and Provocative Question 12 is “What are the molecular and/or cellular mechanisms that underlie the development of cancer therapy-induced severe adverse sequelae?” Through a separate initiative, NCI has joined an NIH-wide program announcement requesting applications for “Serious Drug Reaction Research”. These initiatives support pre-clinical, translational, and clinical research in areas including the mechanisms underlying serious adverse drug reactions (ADRs), including organ toxicities resulting from anti-cancer therapies; and discovery and integration of informative biomarkers for prediction, early detection, or monitoring of ADRs; and development of interventions for alleviation of severe and/or chronic ADRs in cancer patients. A better understanding of the mechanisms underlying of adverse events holds the promise of improving the lives of cancer patients without jeopardizing the effectiveness of their anti-cancer therapies.
